# Absence of lymphogranuloma venereum among men who have sex with men on PrEP receiving doxycycline postexposure prophylaxis

**DOI:** 10.1093/jac/dkaf456

**Published:** 2025-12-16

**Authors:** Cristina Gómez-Ayerbe, Rosario Palacios, Salvador Martín-Cortés, Jesús Santos

**Affiliations:** Infectious Diseases Unit, Hospital Universitario Virgen de la Victoria (Málaga), Spain; IBIMA-Plataforma BIONAND, Málaga, Spain; Infectious Diseases Unit, Hospital Universitario Virgen de la Victoria (Málaga), Spain; IBIMA-Plataforma BIONAND, Málaga, Spain; IBIMA-Plataforma BIONAND, Málaga, Spain; Internal Medicine, Hospital Universitario Virgen de la Victoria (Málaga), Spain; Infectious Diseases Unit, Hospital Universitario Virgen de la Victoria (Málaga), Spain; IBIMA-Plataforma BIONAND, Málaga, Spain

Dear Editor,

Lymphogranuloma venereum (LGV), caused by *Chlamydia trachomatis* (CT) serovars L1–L3, has been increasingly reported among men who have sex with men (MSM), including HIV-negative individuals enrolled in pre-exposure prophylaxis (PrEP) programmes.^1,2^ Although LGV remains uncommon in many settings, its invasive nature and rising prevalence in some MSM populations warrant attention. On the other hand, the use of doxycycline in prevention of sexually transmitted infections (STI) in MSM at risk, is becoming more popular after the publication of different Clinical Trials as Luetkemeyer *et al.* (*NEJM* 2023) or Molina *et al.* (*Lancet Infect Dis* 2018).^3–6^ Even though in all of them, the decrease in the incidence of CT infection is the rule, the impact on the appearance of LGV has not been reported. We have recently communicated our experience in MSM on PrEP who started a doxycycline postexposure prophylaxis (doxy-PEP) programme in real life.^7^ In this ongoing study we wanted to assess the potential impact of doxy-PEP on the incidence of LGV analysing rectal CT diagnosed by real-time PCR (Cobas 4800, Roche^®^) in the same laboratory before and after the introduction of doxy-PEP. From March 2023 to May 2025, we included 305 participants with a mean follow-up time of 18 (**±**12) months before starting doxy-PEP and a mean follow-up time of 15 (**±**7) months after starting doxy-PEP. Follow-up times were between 3 and 6 months for all participants, with same procedures, before and after doxy-PEP beginning. Before doxy-PEP initiation, we identified 94 rectal CT infections (incidence rate 20.6 cases per 100 person-years; 95% CI: 16.6–25.1), of which 19 (20%) were confirmed (by PCR-based genotyping) LGV serovars (incidence rate: 4.15 cases per 100 person-years; 95% CI: 3.75–9.73). Following the introduction of doxy-PEP, 18 rectal CT infections were diagnosed (incidence rate 4.7 cases per 100 person-years; 95% CI: 2.8–7.5), none of which were caused by LGV serovars (Fisher’s exact test, 20% versus 0% *P* = 0.000286) (Table 1). This represents a 77% reduction in CT incidence (incidence rate ratio: 0.23; 95% CI 0.14–0.38; *P* = 0.0001).

**Table 1.** Incidence of CT and LGV before and after doxy-PEP implementation

Infection type	Period	No. of events	Person-years	Incidence (per 100 person-years, 95% CI)	IRR (95% CI)	*P* value
CT	Pre-doxy-PEP	94	457.5	20.6 (16.6–25.1)	—	—
	Post-doxy-PEP	18	381.3	4.7 (2.8–7.5)	0.23 (0.14–0.38)	<0.0001
LGV	Pre-doxy-PEP	19	457.5	4.15 (2.50–6.49)	—	—
	Post-doxy-PEP	0	381.3	0 (0–0.79)	—	0.000003

IRR, incidence rate ratio.

Although these are observational results and do not prove causality, these findings suggest that doxy-PEP may not only reduce overall CT incidence but could also help to prevent more invasive LGV infections. This could have possible public health implications and must be balanced with concerns about antimicrobial resistance and prolonged antibiotic exposure. We also believe that decline in LGV could be related to changes in sexual behaviour, screening frequency or diagnostic practices that were not controlled. However, given the limited sample size that limits the strength of the conclusions, the limited statistical power and the observational nature of our data, caution is warranted. Larger, prospective studies are needed to clarify the role of doxy-PEP in preventing LGV and to better understand the evolving epidemiology of LGV in the context of MSM on PrEP.

Sincerely,
